# Supplementary material for: Quantitative genetic architecture of adaptive phenology traits in the deciduous tree, Populus trichocarpa (Torr. and Gray)
Source: Heredity (Edinb). 2020 Sep 8;125(6):449–58. doi: 10.1038/s41437-020-00363-z (PMC7784687; doi:10.1038/s41437-020-00363-z)
Supplement: Supplementary file 1 — Supplementary material [file 41437_2020_363_MOESM1_ESM.pdf]

Supplementary Files

Figure S1: Climate variation between 1896 and 2019. Black dots represent monthly means, red and blue lines link the monthly means during the experiment scoring period.

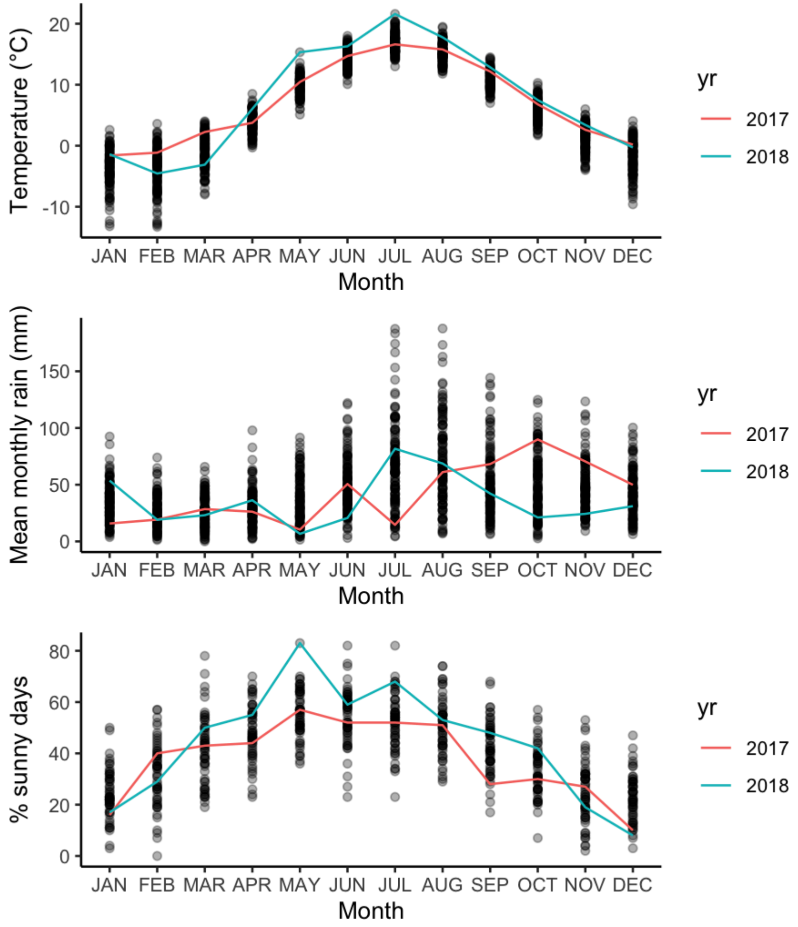

Figure S2: Smoothed fits used to extract transition dates (x axis) for developmental stages (y axis). Random individual trees are shown to illustrate the difference between smoothed/imputed data (orange) and raw data (red). Orange fit produce more reliable data and are used throughout the analyses.

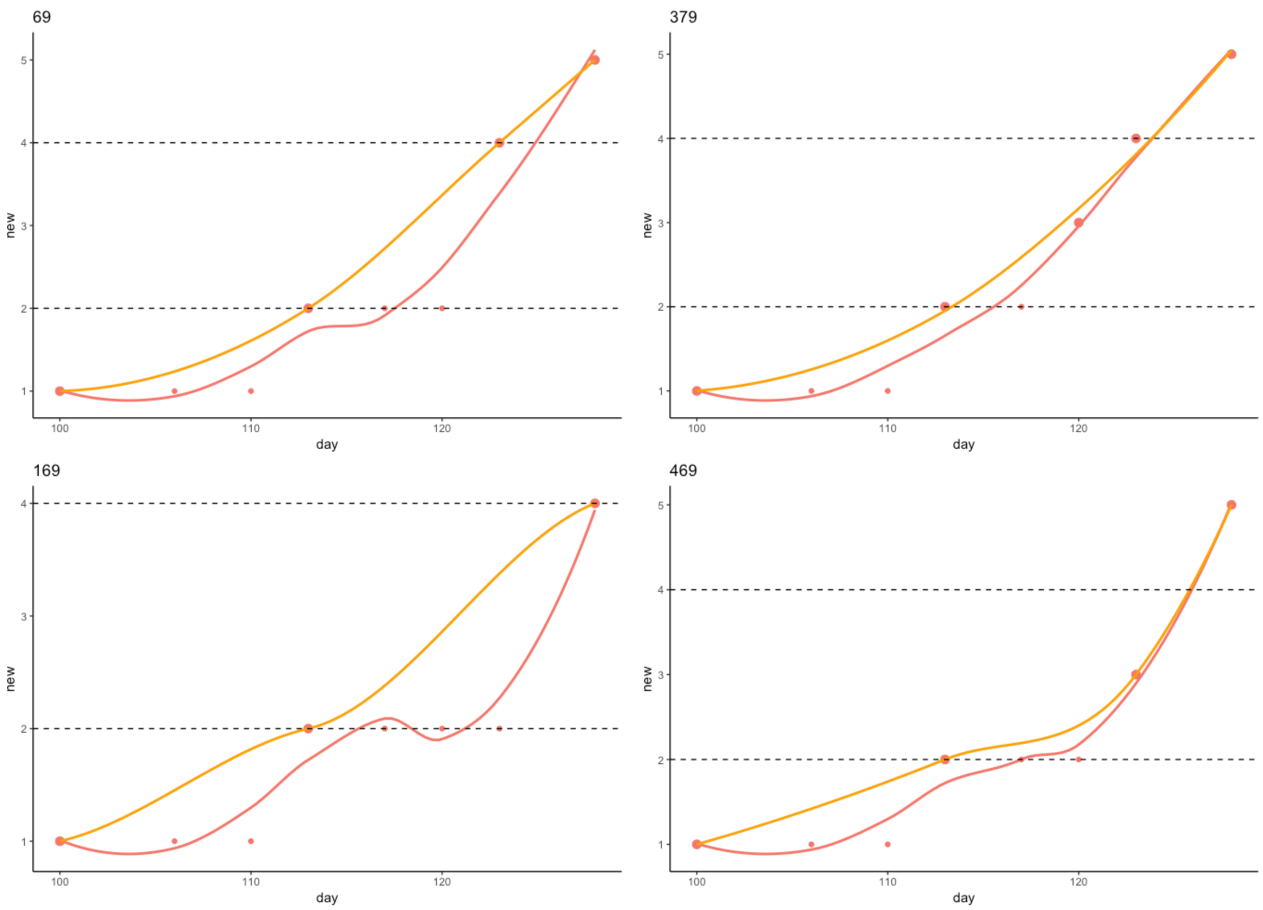

Table S1: Crossing scheme, and parental information for experimental trees

			Fathers									
		Region	Alaska		BC	Idaho	Oregon	Washington x Oregon	Washington	OP, Koster, Holland		-
Mothers		Lat (alt.)	58°30' (15)	60°00'	49°15'	48°25'	45°35'		45°50' (75)			
Region	Lat (alt.)	Klon	**293**	**278**	**193**	214	159	236	**299**	187	209	290
Alaska	58°30'	**216**			5		1		5			5
	59°20' (500)	**291**						2		1		
	60°37' (400)	**265**	3	3		1			2	2		
BC	49°00'	**230**	7	4		3		2	6			
	-	96							1			
Idaho x Oregon	-	237	2	1		3			3	1		3
	-	**240**		4				1	21		2	
Oregon	44°30'	271	1	2				1	4			1
-		301								2		1
